# Allyl ether of mansonone G as a potential anticancer agent for colorectal cancer

**DOI:** 10.1038/s41598-022-23997-x

**Published:** 2022-11-16

**Authors:** Savinee Chanvijit, Suttinee Phuagkhaopong, Panupong Mahalapbutr, Methus Klaewkla, Warinthorn Chavasiri, Piyanuch Wonganan

**Affiliations:** 1 Interdisciplinary Program in Pharmacology, Graduate School, Chulalongkorn University, Bangkok, 10330 Thailand; 2 Department of Pharmacology, Faculty of Medicine, Chulalongkorn University, 1873 Rama IV Road, Pathumwan, Bangkok, 10330 Thailand; 3 Department of Biochemistry, Faculty of Medicine, Khon Kaen University, Khon Kaen, 40002 Thailand; 4 Future Health Innovation Technology Co., Ltd., Bangkok, 10170 Thailand; 5 Center of Excellence in Natural Products Chemistry, Department of Chemistry, Faculty of Science, Chulalongkorn University, Bangkok, 10330 Thailand

**Keywords:** Pharmacology, Cell biology, Drug discovery

## Abstract

Mansonone G (MG), a 1,2-naphthoquinone isolated from the heartwood of *Mansonia gagei* Drumm, exhibited several pharmacological activities such as anti-bacterial, anti-estrogenic and anti-adipogenic effect. This study evaluated the cytotoxicity of MG and its derivatives as well as determined the mechanism(s) underlying the cytotoxic activity of the most potent MG derivative on two CRC cell lines, HCT-116 cells carrying p53 wild-type and HT-29 cells carrying p53 mutant. We found that MG and its derivatives could inhibit viability of HCT-116 and HT-29 cells in a concentration-dependent manner. Of all semi-synthetic derivatives of MG, allyl ether mansonone G (MG7) was the most potent cytotoxic agent toward cancer cells and less toxic to normal cells. MG7 could induce ROS generation which was associated with cytotoxicity and apoptosis in both HCT-116 and HT-29 cells. Western blot analysis revealed that MG7 downregulated the expression of Bcl-2 and Bcl-xL proteins in both CRC cell lines and upregulated the expression of BAK protein in HT-29 cells. Moreover, MG7 inhibited AKT signaling pathway in both CRC cell lines and modulated ERK1/2 signaling pathway by inhibiting ERK1/2 phosphorylation in HCT-116 cells and activating ERK1/2 phosphorylation in HT-29 cells. Molecular docking revealed that MG7 could bind to the ATP-binding pocket of AKT and ERK1 via hydrophobic interactions.

## Introduction

Colorectal cancer (CRC) is one of the most common cancer worldwide^1^. Treatment options of CRC are surgery, radiotherapy, chemotherapy and targeted therapy. The drugs commonly used for the treatment of CRC are 5-fluorouracil (5-FU), oxaliplatin and irinotecan^2^. Although chemotherapy has been widely used, it has many side effects which can affect the treatment for CRC. Moreover, CRC can become resistant to chemotherapy rendering it useless. Therefore, new compounds with potent anticancer activity are urgently needed.

*Mansonia gagei* Drumm, commonly found in tropical countries such as Myanmar and Thailand, has been locally used as an antidepressant, antiemetic, cardiac stimulant and refreshment agents^3^. Several previous studies reported that mansonone G, a major compound isolated from the heartwood extract of *Mansonia gagei* Drumm, has various biological activities such as antibacterial, antioxidant, antiestrogenic, anticancer and antifungal effect^3–6^. Remarkably, many semi-synthetic derivatives of MG have shown to exhibit greater pharmacological activities than the parent compound. Allyl and phenyl ethers of MG have demonstrated antibacterial activity against *Staphylococcus aureus* 64 times higher than the parent MG compound^3^. In addition, recent studies have reported that ethoxy and butoxy MGs were more cytotoxic to non-small cell lung cancer and breast cancer cells than their parent compound^7, 8^. Therefore, the present study assessed the cytotoxic activity of MG and its ether analogs on two human colorectal cancer cells lines, HCT-116 cells carrying p53 wild-type and HT-29 cells carrying p53 mutant.

## Results

### Effect of MG and its derivatives on the viability of CRC cells

**Figure 1:**
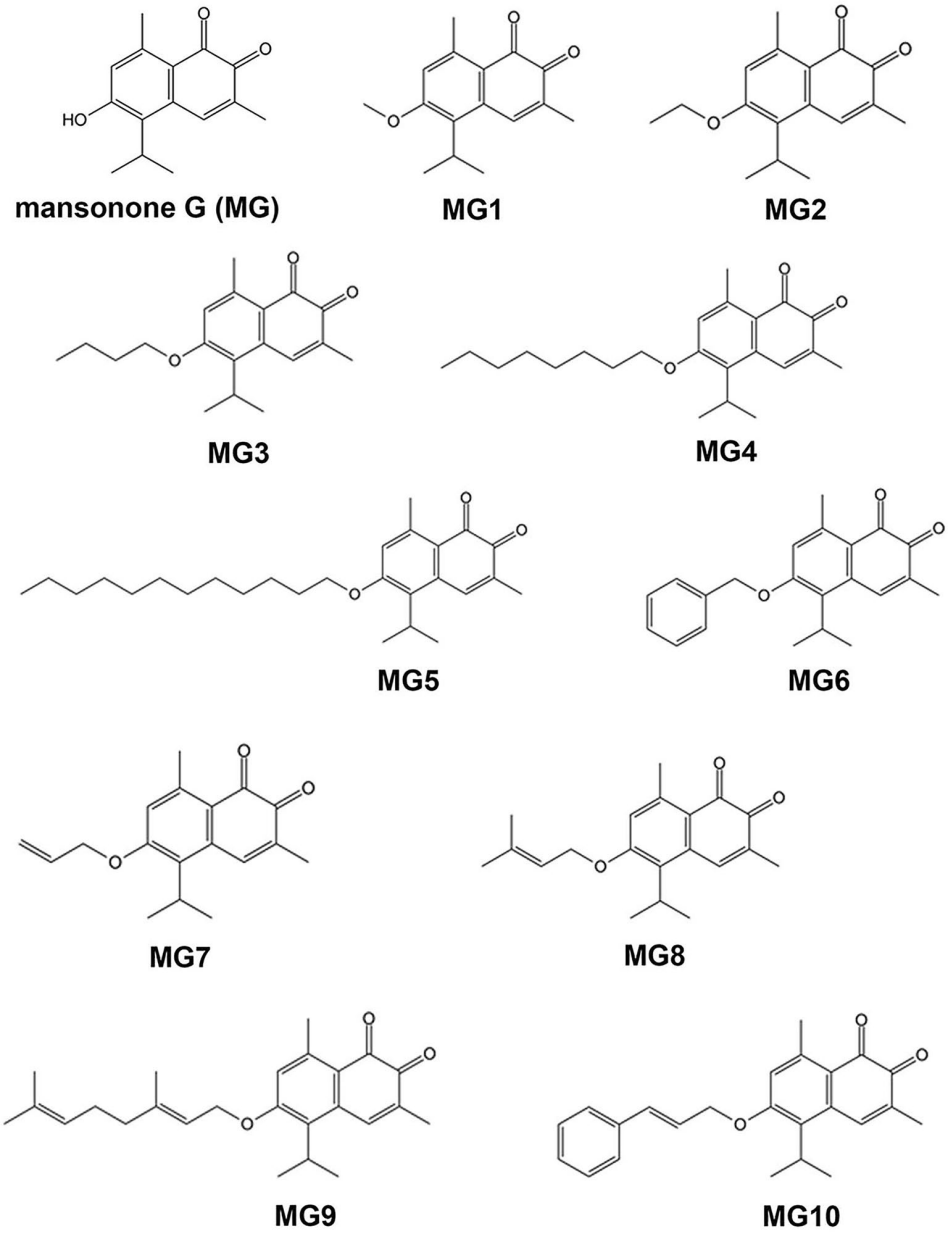
Chemical structures of MG and its semisynthetic derivatives.

**Figure 2:**
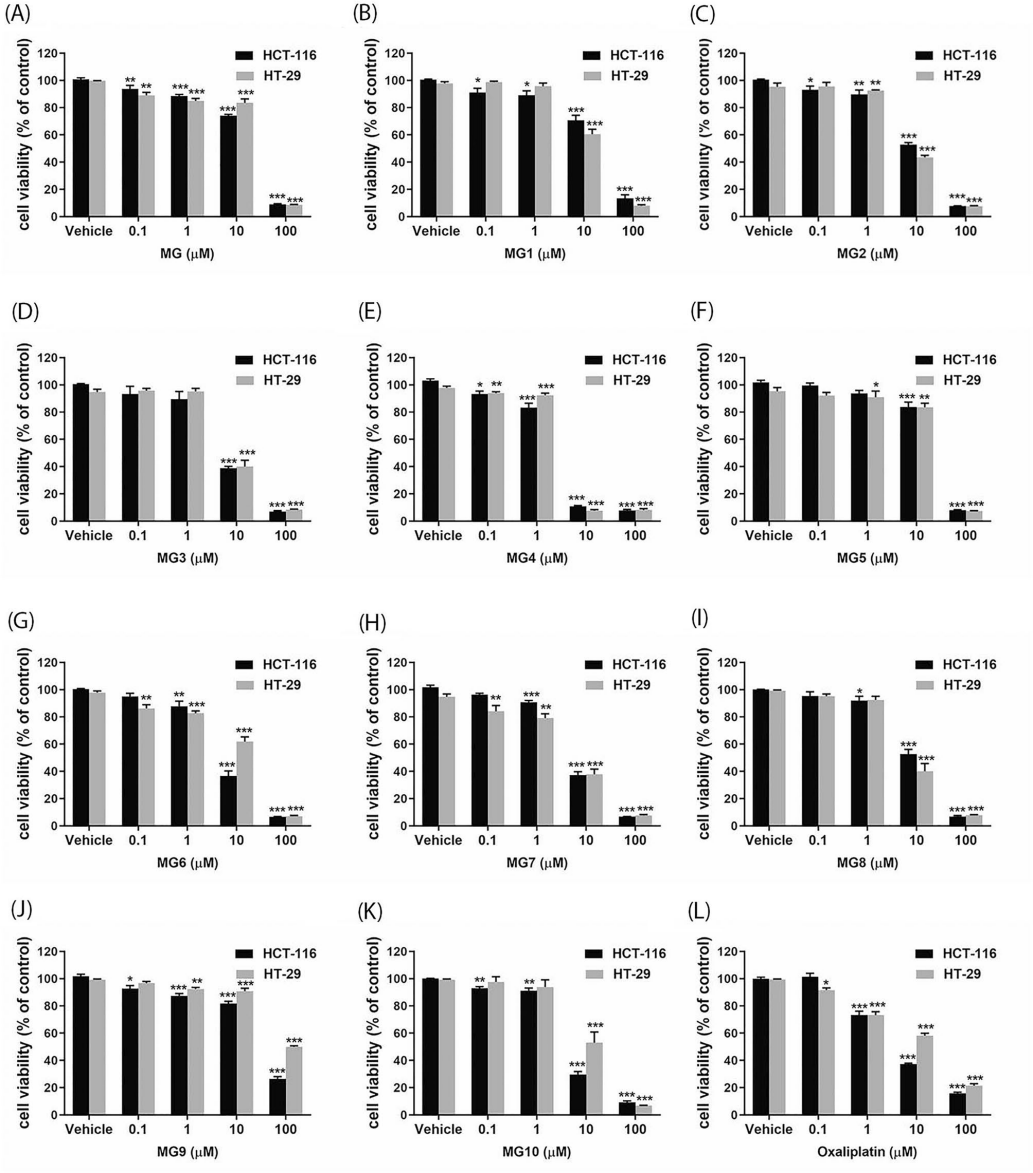
Effects of MG and its derivatives on viability of (**A**) HCT-116 and (**B**) HT-29 cells. The cells were treated with MG, semi-synthesized derivative of MGs or oxaliplatin at 0.1, 1, 10, and 100 µM for 48 h. Cell viability was evaluated using MTT assay. Data are presented as mean ± S.E.M of three independent experiments. *P < 0.05, **P < 0.01 and ***P < 0.001 compared to vehicle control (0.2% DMSO).

Initially, the cytotoxic activity of MG and ten semi-synthesized derivatives (Fig. 1) were determined using p53 wild-type HCT-116 cells and p53 mutant HT-29 cells. Oxaliplatin was used as the positive control. As shown in Fig. 2A–L, MG and its derivatives as well as oxaliplatin significantly inhibited the growth of the two CRC cell lines in a concentration-dependent manner (*P* < 0.05). Remarkably, all semi-synthesized MGs, except MG5 and MG9, were more cytotoxic to cancer cells than the parent MG compound (Table 1). Of all MG derivatives, MG4 exhibited the most potent cytotoxicity toward HCT-116 cells, followed by MG10, MG6 and MG7. Similarly, MG4, MG7, MG8 and MG3 displayed high cytotoxic activities against HT-29 cells. Therefore, MG derivatives had a strong anticancer activity towards both CRC cell lines (IC_50_ < 10 µM). As a result of this, MG3, MG4, MG7 and MG10 were selected for toxicity testing using PCS201-010 and CRL-1790 cells.

**Table 1:** IC_50_ values of MG and its derivatives on HCT-116 and HT-29 cells.

Compounds	IC_50_ (µM)	
	HCT-116	HT-29
MG	20.74 ± 0.51	25.55 ± 2.08
MG1	20.86 ± 3.10	15.07 ± 1.91
MG2	10.60 ± 0.65	8.43 ± 0.81
MG3	6.63 ± 0.60	8.09 ± 1.13
MG4	2.77 ± 0.20	3.24 ± 0.21
MG5	26.59 ± 2.39	26.68 ± 2.08
MG6	6.13 ± 0.59	13.62 ± 1.42
MG7	6.54 ± 0.47	5.66 ± 1.13
MG8	10.83 ± 1.44	7.53 ± 1.20
MG9	37.85 ± 1.42	98.59 ± 3.37
MG10	5.16 ± 0.53	9.89 ± 1.49
Oxaliplatin	4.61 ± 0.22	12.70 ± 1.72

### Effect of MG derivatives on the viability of PCS201-010 and CRL-1790 cells

**Figure 3:**
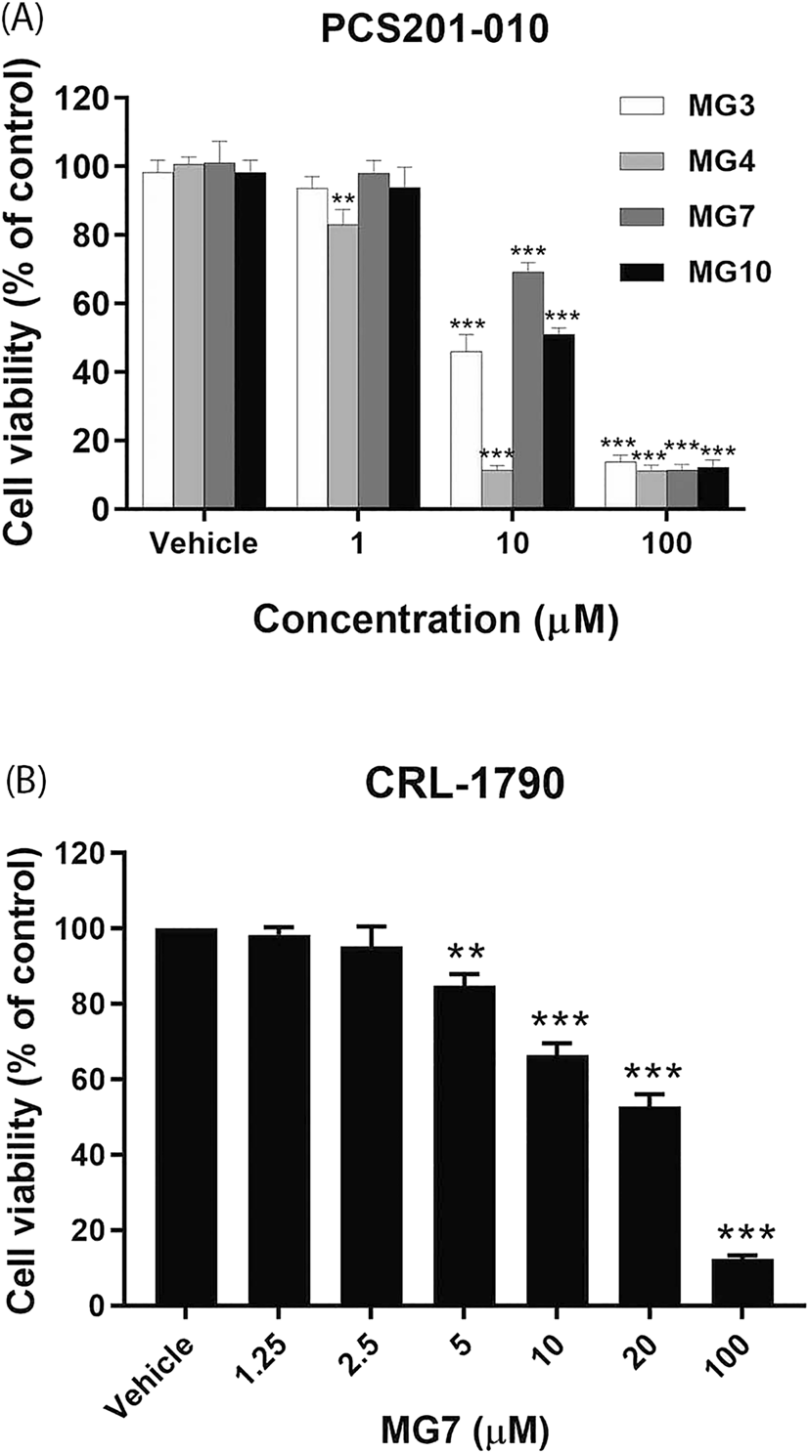
Effects of semi-synthetic derivatives of MGs on viability of (**A**) PCS201-010 and (**B**) CRL-1790 cells. The cells were treated with MG3, MG4, MG7 or MG10 at indicated concentrations for 48 h. Cell viability was evaluated using MTT assay. Data are presented as mean ± S.E.M of three independent experiments. **P < 0.01 and ***P < 0.001 compared to vehicle control (0.2% DMSO).

We further evaluated the toxicity of our selected MGs on PCS201-010 normal human dermal fibroblast cells. As shown in Fig. 3A, all four tested compounds decreased the viability of PCS201-010 in a concentration-dependent manner. However, it should be noted that MG7 was relatively less toxic to normal fibroblast cells. To confirm whether MG7 (allyl ether of mansonone G) was not too toxic to normal cells, cytotoxicity of MG7 was also evaluated on CRL-1790 normal human colon epithelial cells. The obtained IC_50_ value was 22.07 ± 1.10 µM (Fig. 3B), which was approximately three times higher than those found in CRC cells. Given that MG7 was more toxic to CRC cells than normal colon cells, anticancer activity and the underlying mechanisms of MG7 were further explored.

### Effect of allyl ether mansonone G on apoptosis induction in CRC cells

**Figure 4:**
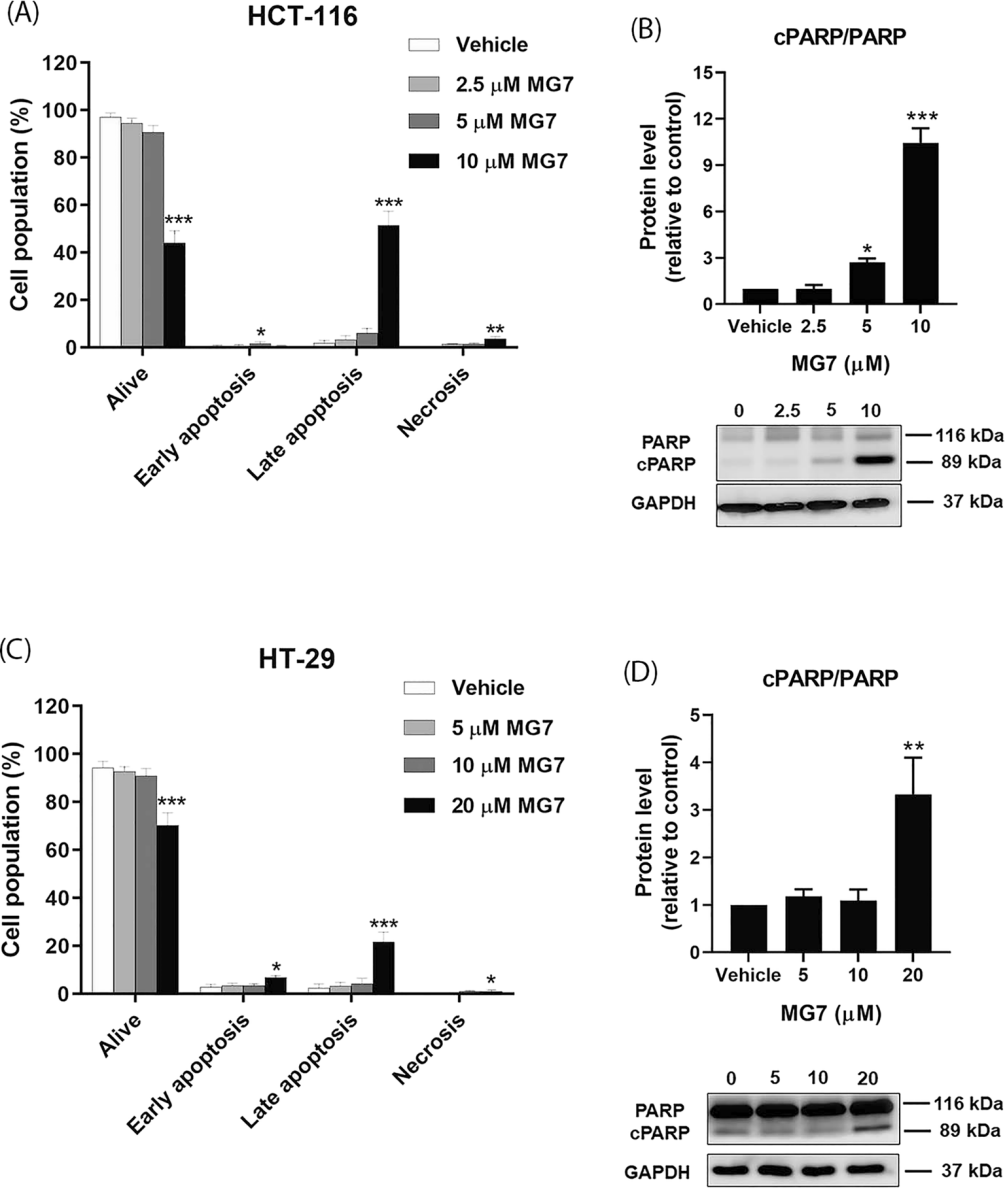
Effects of MG7 on apoptosis induction in CRC cells. (**A**) The percentages of apoptotic HCT-116 cells after being treated with MG7 at 2.5, 5 and 10 µM for 24 h were evaluated using annexin-V/PI staining followed by flow cytometry. (**B**) The expression ratio of cleaved PARP to total PARP proteins in HCT-116 cells was determined using western blot. (**C**) The percentage of apoptotic HT-29 cells and (**D**) the expression ratio of cleaved PARP to total proteins in HT-29 cells after being treated with MG7 at 5, 10 and 20 µM for 24 h. *P < 0.05, **P < 0.01 and ***P < 0.001 compared to vehicle control (0.2% DMSO).

To determine whether cytotoxicity of MG7 in CRC cells is mediated through apoptosis induction, flow cytometric analysis of annexin V-FITC/PI stained cells was performed. As shown in Fig. 4A, in HCT-116 cells, there was a significant increase in the percentage of early apoptotic cells that was detected after treatment with 5 µM of MG7. Remarkably, MG7 at 10 µM significantly induced HCT-116 cells to undergo late apoptosis and necrosis was approximately 20- to 7-fold higher than the control. The results from the flow cytometry was in agreement with the findings from the western blot. The results from the western blot revealed that the treatment of HCT-116 cells with MG7 at 5 and 10 µM significantly induced the cleavage of poly(ADP-ribose) polymerase (PARP) (Fig. 4B). Similar results were detected in HT-29 cells treated with MG7 at 20 µM. The numbers of early and late apoptotic cells were increased by approximately 2 and 6 times compared to the vehicle control (Fig. 4C). A significant increase in cleaved PARP protein level was also detected in HT-29 cells in response to 20 µM of MG7 treatment (Fig. 4D).

### Effect of allyl ether mansonone G on the expression of Bcl-2 family proteins in CRC cells

**Figure 5:**
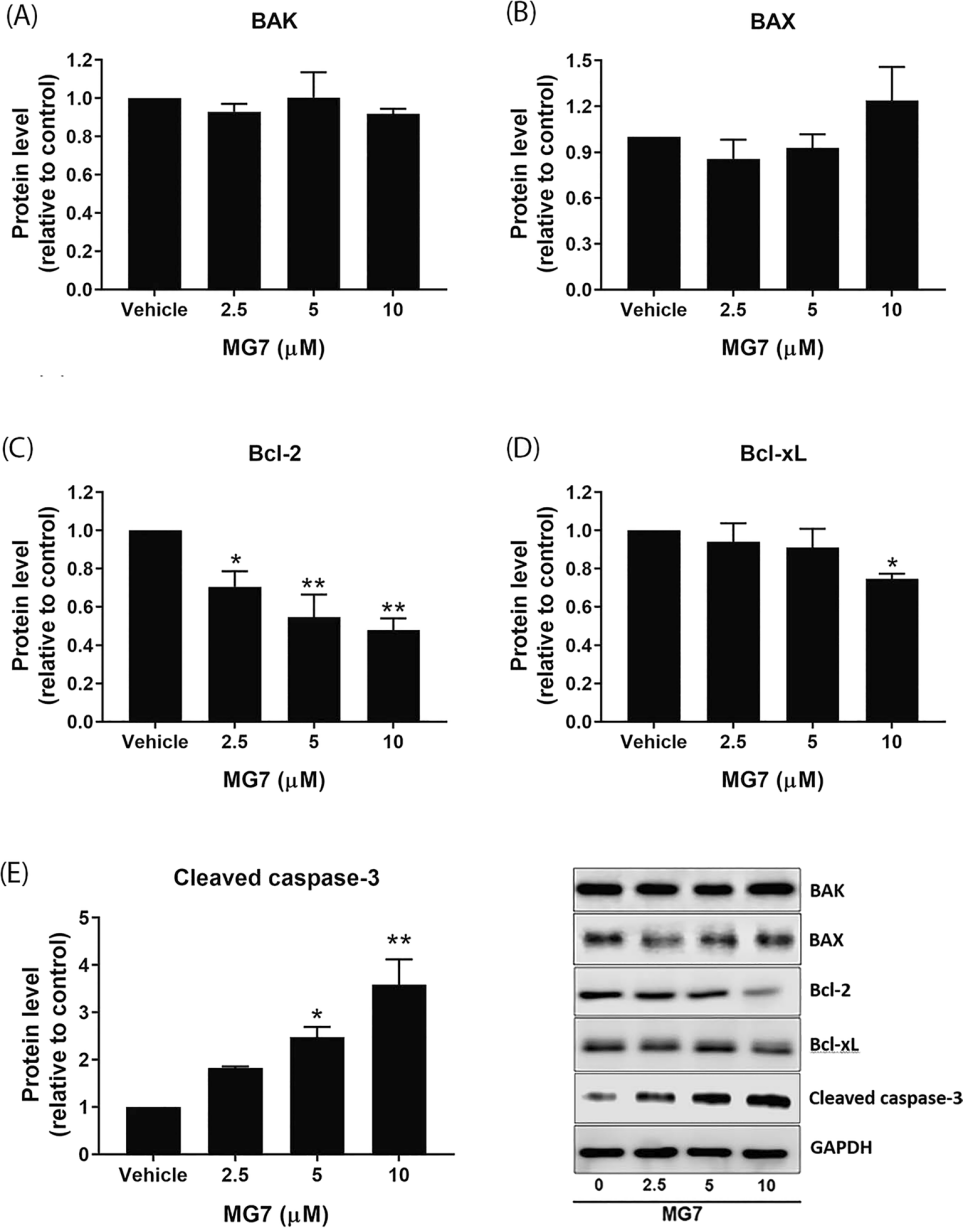
Effects of MG7 on the protein expression of Bcl-2 family members in HCT-116 cells. The cells were treated with MG7 at concentrations of 2.5, 5, and 10 µM for 24 h. The expression levels of (**A**) BAK, (**B**) BAX, (**C**) Bcl-2, (**D**) Bcl-xL and (**E**) cleaved caspase-3 proteins were determined using western blot. Data are expressed as mean ± SEM of three independent experiments. *P < 0.05 and **P < 0.01 compared to vehicle control (0.2% DMSO).

**Figure 6:**
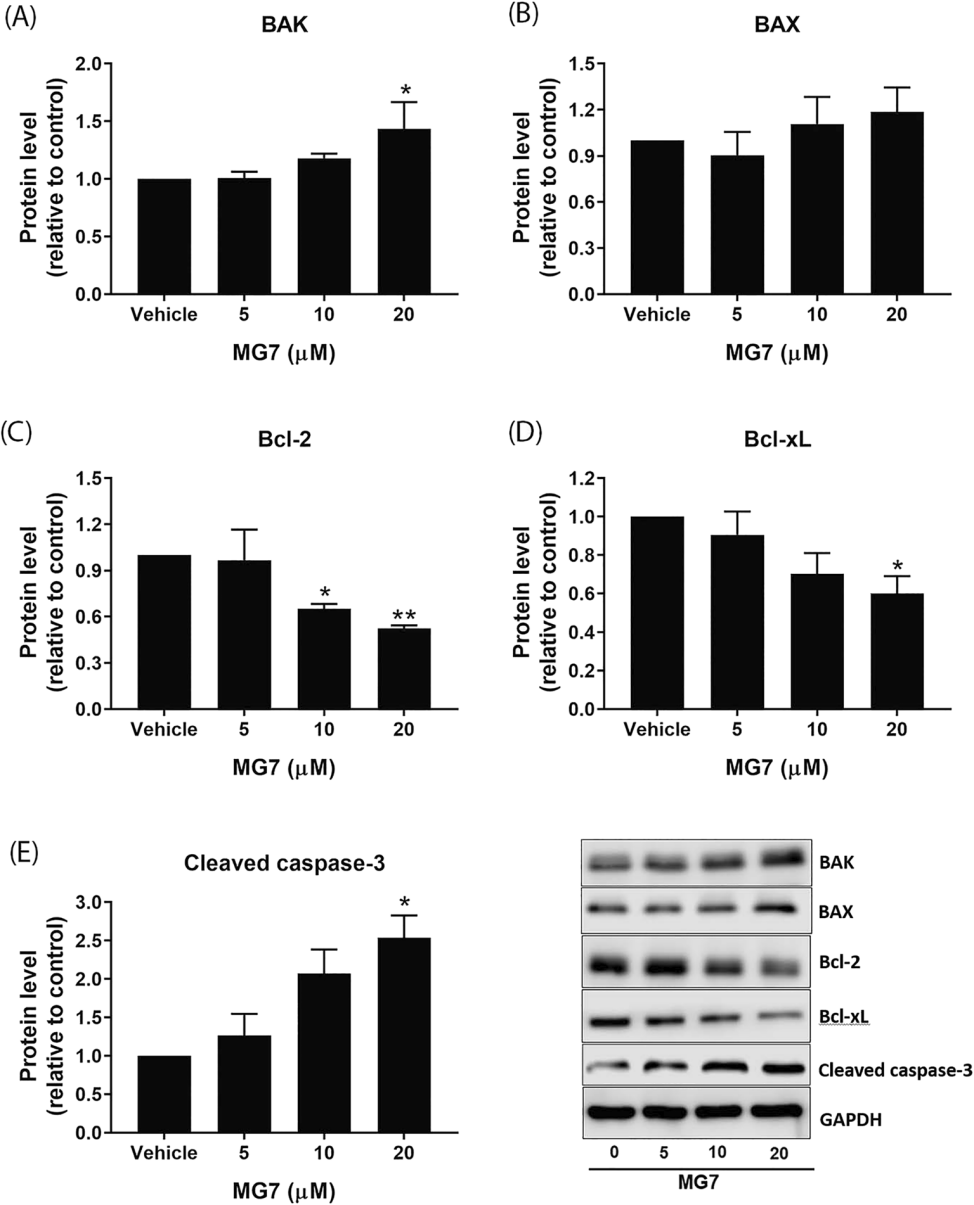
Effects of MG7 on the protein expression of Bcl-2 family members in HT29 cells. The cells were treated with MG7 at concentrations of 5, 10 and 20 µM for 24 h. The expression levels of (**A**) BAK, (**B**) BAX, (**C**) Bcl-2, (**D**) Bcl-xL and (**E**) cleaved caspase-3 proteins were determined using western blot. Data are expressed as mean ± SEM of three independent experiments. *P < 0.05 and **P < 0.01 compared to vehicle control (0.2% DMSO).

To investigate whether apoptosis-inducing effect of MG7 is mediated through modulation of Bcl-2 family members, the expression levels of pro-apoptotic proteins, BAK and BAX, and anti-apoptotic proteins, Bcl-2 and Bcl-xL were analyzed. As shown in Fig. 5A,B, treatment of HCT-116 cells with MG7 did not alter the expression of BAK and BAX. However, it should be noted that MG7 significantly suppressed the expression of Bcl-2 protein in a concentration-dependent manner (Fig. 5C, *P* < 0.05). Similarly, there was a significant decrease in Bcl-xL protein level in HCT-116 cells treated with 10 µM of MG7 (Fig. 5D, *P* < 0.05). To further confirm the apoptosis-inducing effect of MG7 on HCT-116 cells, the cleavage of caspase-3, a key hallmark of apoptosis, was evaluated. As shown in Fig. 5E, MG7 at 5 and 10 µM significantly induced the protein expression of cleaved caspase-3. In HT-29 cells, the expression of BAK, a pro-apoptotic protein, was significantly upregulated after treatment with 20 µM of MG7 (Fig. 6A, *P* < 0.05) whereas the expression of BAX was not affected by MG7 (Fig. 6B). Aside from BAK, MG7 at 10 and 20 µM dramatically downregulated Bcl-2 protein levels in HT-29 cells by 35% and 47%, respectively (Fig. 6C). We also observed a significant decrease in Bcl-xL protein level to approximately 40% in HT-29 cells after treatment with 20 µM of MG7 (Fig. 6D). Similarly, MG7 at 20 µM significantly increased cleaved caspase-3 protein (Fig. 6E, *P* < 0.05).

### ROS mediates allyl ether manosone G-induced apoptosis in CRC cells

**Figure 7:**
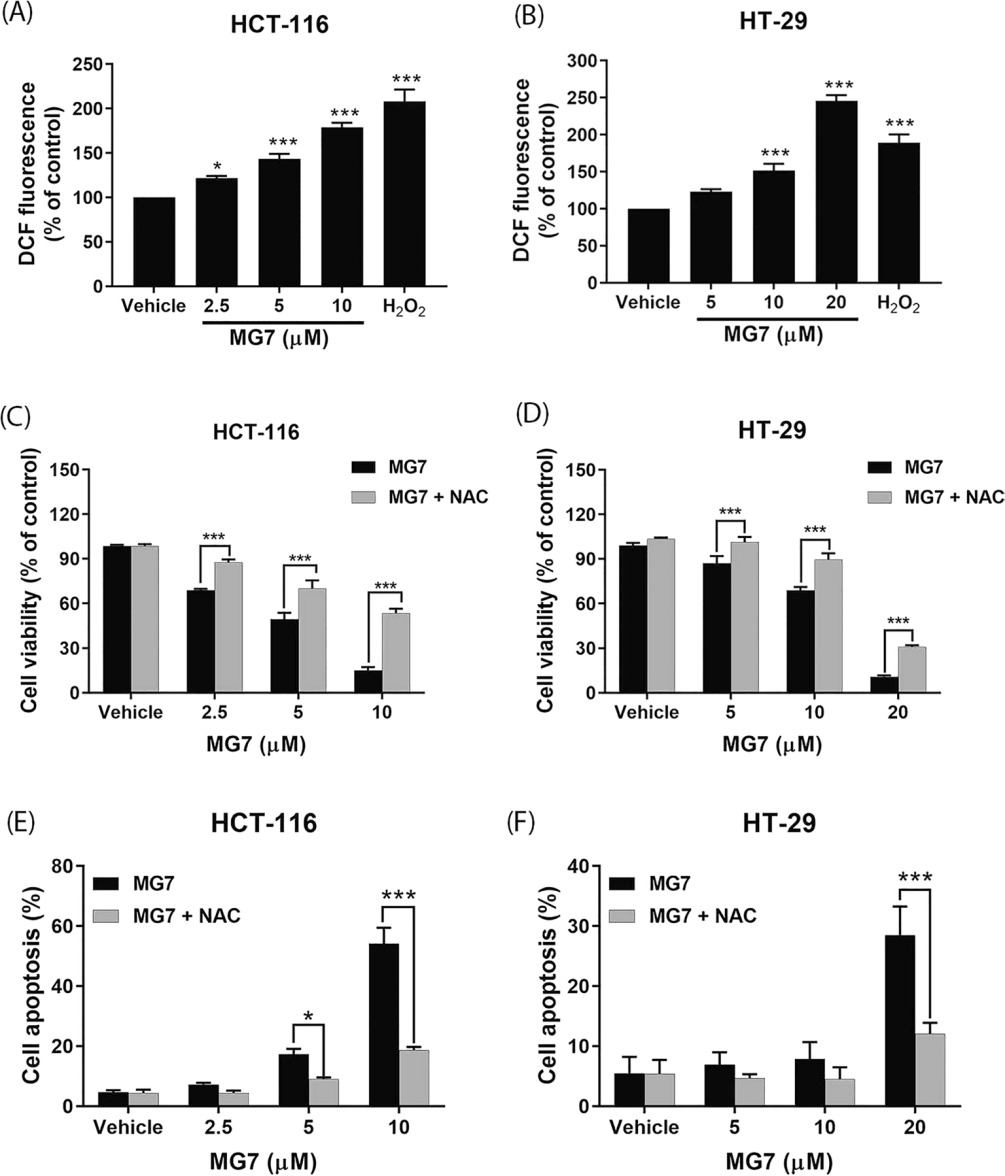
Roles of ROS on cytotoxic and apoptosis-inducing effects of MG7. The ROS levels in (**A**) HCT-116 and (**B**) HT-29 cells after being treated with MG7 at indicated concentrations or H_2_O_2_ at 200 µM were evaluated using DCFH_2_-DA assay. The viability of (**C**) HCT-116 and (**D**) HT-29 cells and the numbers of annexin V-positive apoptotic (**E**) HCT-116 and (**F**) HT-29 cells after being treated with MG with or without NAC. *P < 0.05, **P < 0.01 and ***P < 0.001 compared to vehicle control (0.2% DMSO).

We then determined whether cytotoxic and apoptosis-inducing effects of MG7 are mediated through the generation of ROS in CRC cells. As illustrated in Fig. 7A, MG7 significantly increased ROS levels in HCT-116 cells in a concentration-dependent manner (*P* < 0.05). Similarly, in HT-29 cells, the treatment with MG7 at 10 and 20 µM markedly increased ROS levels by approximately 1.5 and 2.4 times, respectively, compared to the levels seen after treatment with the vehicle control (Fig. 7B, *P* < 0.001). We then determined whether the generation of ROS is involved in the cytotoxic effect of MG7. The CRC cells were incubated with 5 mM of *N*-acetylcysteine (NAC), a ROS scavenger, for 2 h followed by MG7 treatment for 24 h. The MTT results revealed that NAC pretreatment significantly reduced cytotoxicity of MG7 in both HCT-116 and HT-29 cells (Fig. 7C,D). Moreover, the pretreatment with NAC significantly decreased the numbers of apoptotic cells by approximately 2 and 3 times compared to treatment with MG7 at 5 and 10 µM alone, respectively (Fig. 7E, *P* < 0.05). Like HCT-116 cells, MG7 at 20 µM significantly induced apoptosis in HT-29 cells and NAC pretreatment obviously decreased the percentages of apoptotic cells by 2.4-fold (Fig. 7F, *P* < 0.001).

### RNA-seq transcriptome analysis of CRC cells under allyl ether mansonone G

**Figure 8:**
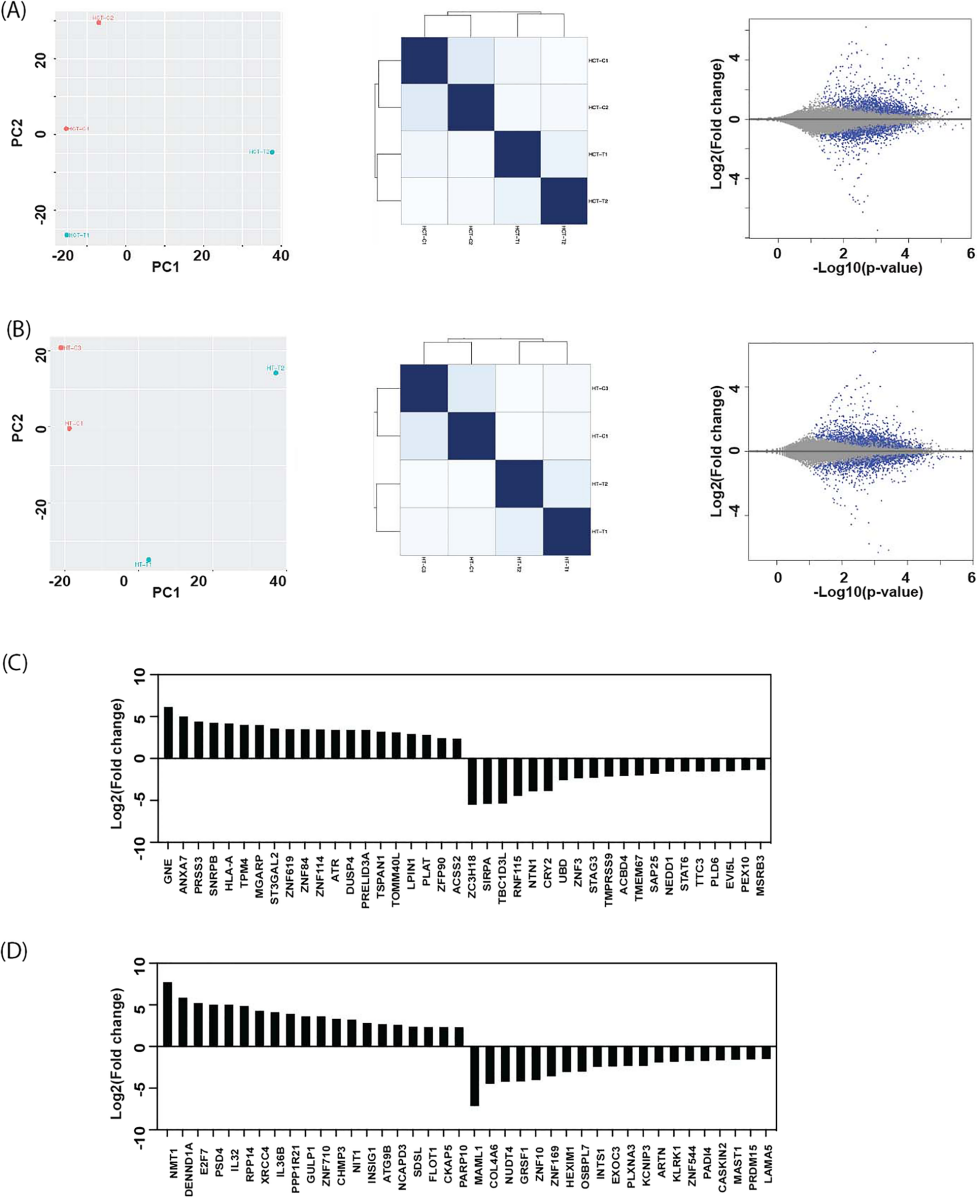
Comprehensive transcriptome dynamics by the MG7 treatment in HCT-116 and HT-29 cells. Principal-component analysis (PCA) of directional RNA-seq data (left panel), heat map and hierarchical clustering of transcriptional pattern analysis (middle panel), and volcano plot indicating differentially expressed transcripts (right panel) after MG7 treatment by HCT-116 cells (**A**) and HT-29 cells (**B**). Heatmap describing fold changes for top 20 up-regulated and down-regulated transcripts indicating statistically significant differences between the MG7-treated group and the control group in HCT-116 (**C**) and HT-29 cells (**D**).

To compare the effects of MG7 exposure on transcriptional changes in two different types of colorectal cancer cells; HCT-116 and HT-29 cells, the gene expression profiling was performed using RNA-seq. The principal component analysis (PCA) on transcripts and hierarchical clustering of transcriptional pattern analysis using DEseq2 showed a significant shift in the MG7-treated group (Fig. 8A,B). Volcano plot were generated to show the differential expression pattern of entire mRNA transcripts during exposure to MG7 in HCT-116 and HT-29 cells. Among annotated 2486 genes in HCT-116 and 2425 genes in HT-29, 142 were up-regulated and 175 were down-regulated in HCT-116, and 146 were up-regulated and 129 were down-regulated in HT-29 (Fig. 8A,B). Top 20 (up and down-regulated) differentially expressed genes (DEGs) between control and MG-7 treatment groups in HCT-116 and HT-29 cells were illustrated in Fig. 8C,D. The results showed that MG7 affected expression of transcripts differently among p53 wild type (HCT-116) and p53 mutant (HT-29) colorectal cancer cells (Tables S1 and S2). To identify the enriched biological function and pathway affected by MG7 treatment, the up- and down-regulated transcripts influenced by the treatment were independently evaluated using functional enrichment analysis (G0:BP) following by KEGG enrichment analysis. The results indicated that MG7 caused cytotoxicity and apoptosis in colorectal cancer cells by mediated the expression of gene involved in cell cycle, apoptosis and necroptosis through key anticancer pathways such as NF-kappa B, Ras, MAPK, phosphatidylinositol, and mTOR (Tables S3–S6). In particular, the results of RNA-seq highlighted that MAPK, PPAR and p53 signaling pathways and glutathione metabolism inhibited cell growth and induced apoptosis in HCT-116 cells, whereas PI3K-Akt signaling pathway mainly involved in HT-29 cells.

### Effect of allyl ether mansonone G on MAPK and PI3K/AKT signaling pathways in CRC cells

**Figure 9:**
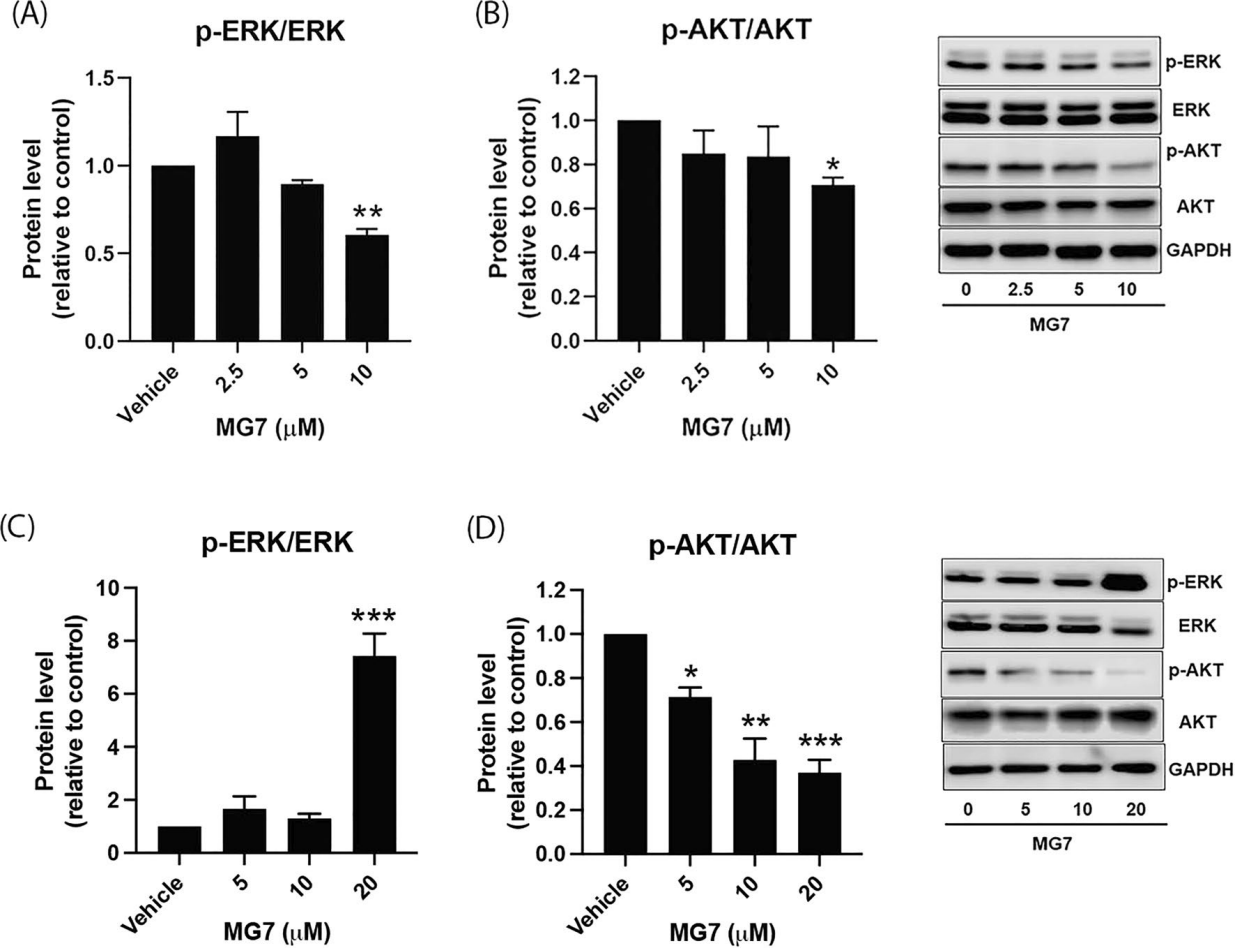
Effects of MG7 on ERK/MAPK and PI3K/AKT signaling pathways in HCT-116 and HT29 cells. The expression ratio of phosphorylated ERK to total ERK and phosphorylated AKT to total AKT in (**A**,**B**) HCT-116 and (**C**,**D**) HT-29 cells after being treated with MG7 at indicated concentrations for 24 h were determined using western blot. *P < 0.05, **P < 0.01 and ***P < 0.001 compared to vehicle control (0.2% DMSO).

To elucidate the effects of MG7 on MAPK/ERK and PI3K/AKT signaling pathways, western blot analysis was performed. In HCT-116 cells, MG7 at 10 µM significantly inhibited the phosphorylation of ERK1/2 and AKT by approximately 40% and 30%, respectively (Fig. 9A,B, *P* < 0.05). In contrast to HCT-116 cells, we observed a significant increase in ERK1/2 phosphorylation in HT-29 cells after treatment with 20 µM of MG7 (Fig. 9C, *P* < 0.001). The expression level of phosphorylated ERK1/2 protein in MG7-treated cells was approximately 7 times higher compared to the vehicle control. It is nevertheless noteworthy that MG7 significantly inhibited AKT phosphorylation in HT-29 cells in a concentration-dependent manner (Fig. 9D, *P* < 0.05).

**Figure 10:**
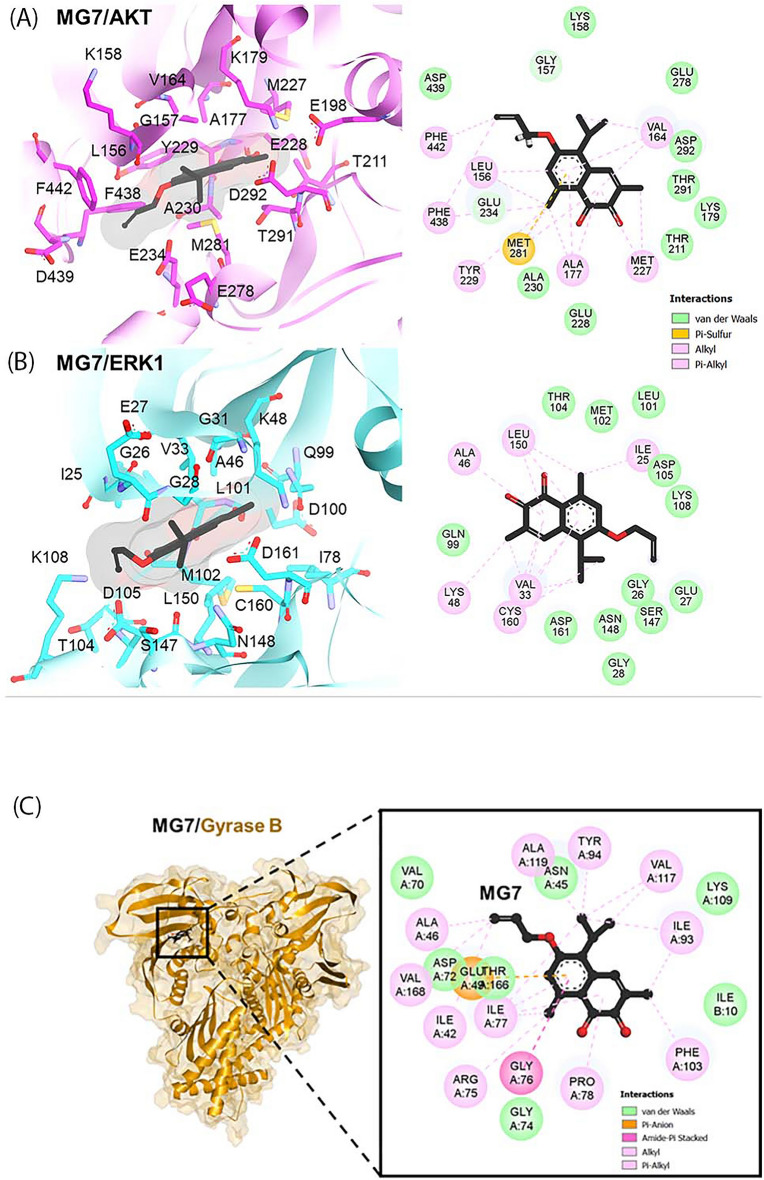
Predicted binding mode of MG7 against (**A**) AKT and (**B**) ERK1 signaling proteins and (**C**) ATPase domain of bacterial DNA gyrase obtained from molecular docking.

We then further investigated the binding pattern of MG7 against these two targets using molecular docking simulation. In the case of MG7/AKT complex, as expected, the hydrophobic MG7 ligand was stabilized mainly by van der Waals and alkyl interactions without forming hydrogen bonding and electrostatic attraction. Notably, a strong pi-sulfur interaction was formed between the aromatic naphthoquinone ring of MG7 and the sulfur atom of Met281 residue of AKT (Fig. 10A). Similarly, the hydrophobic interactions were also the main forces stabilizing MG7/ERK1 complex, and the methyl group at the quinone ring of MG7 formed alkyl interaction with the catalytic Lys48 residue of ERK1 (Fig. 10B). These finding suggested that MG7 could interact with the AKT and ERK1 signaling proteins at the ATP-binding pocket via hydrophobic interactions. Previously, MG7 has been shown to exhibit high antibacterial activity^3^. Thus, in the present study, we have additionally performed the molecular docking simulation to explore the possible binding conformation between MG7 and the bacterial enzyme. As shown in Fig. 10C, MG7 could bind to the ATPase domain of DNA gyrase, an essential bacterial enzyme involved in the DNA replication process^9^, via several hydrophobic interactions.

## Discussion

Mansonones, naphthoquinone-containing compounds isolated from *Mansonia gagei* Drumm, a Thai traditional plant is locally used as an antidepressant, antiemetic, cardiac stimulant and refreshment agent^3^. The present study showed that mansonone G (MG) and its derivatives inhibited the growth of two human CRC cell lines, HCT-116 cells carrying p53 wild-type and HT-29 cells carrying p53 mutant, in a concentration-dependent manner. All semi-synthetic derivatives of MGs, except MG5 and MG9, were more toxic to the cancer cells than the parent compound. These findings were consistent with the finding of Hairani et al., that ether analogues of MG exerted higher antibacterial activity than the parent MG compound^3^. It was suggested that increasing the alkyl chain length of ether analogues of MG made the compounds more hydrophobic which may facilitate the access to bacterial cell wall, resulting in higher antibacterial activity^3^. Therefore, it is possible that increasing the number of carbon units in the alkyl side chain contributes to an increasing cytotoxicity of MG derivatives toward human CRC cells.

Several chemotherapy agents and naphthoquinone-containing compounds induced cancer cells to undergo apoptosis^10–13^. Gene expression profiling by RNA-seq indicated that MG7 caused cytotoxicity and apoptosis in colorectal cancer cells by mediated the expression of gene involved in apoptosis. A previous study conducted in human cervical cancer cells demonstrated that the apoptosis was induced by mansonone E; BAX was upregulated, and Bcl-2 and Bcl-xL were downregulated^5^. In this study, allyl ether mansonone G (MG7), one of the most potent semi-synthesized MGs, could induce apoptosis in both HCT-116 and HT-29 cells. Results from the western blot revealed that MG7 downregulated the expression of Bcl-2 and Bcl-xL proteins in HCT-116 cells whereas in HT-29 cells treated with MG7 upregulated BAK, and downregulated Bcl-2 and Bcl-xL. Taken together, these results suggest that MG7-induced apoptosis is mediated through modulation of Bcl-2 family proteins in HCT-116 and HT-29 cells. It is commonly known that caspase-mediated apoptotic cell death is accomplished through the cleavage of several key proteins required for cellular functions and cell survival. PARP is one of the several substrates of caspases. The present study demonstrated that MG7 induced the cleavage of caspase-3 and PARP in both HCT-116 and HT-29 cells, suggesting that apoptosis-inducing effect of MG7 may be associated with the caspase activation and the cleavage of PARP. Notably, this study found that apoptosis-inducing effect of MG7 was more pronounced in HCT-116 cells than HT29 cells. The results of this study were in agreement with previous studies that cells expressing mutant p53 were more resistant to irradiation, doxorubicin and cisplatin-induced apoptosis than cells expressing wild-type p53^14, 15^.

Compared to PCS201-010 and CRL-1790 cells, cancer cells have higher metabolism and oxidative stress, suggesting that higher levels of ROS can be generated in cancer cells than in PCS201-010 and CRL-1790 cells. Therefore, the accumulation of ROS can be a strategy to selectively kill cancer cells^16^. Previously, naphthoquinone-containing compounds such as curcumin, plumbagin, shikonin and lawsone were shown to induce apoptosis via ROS generation in various cancer cell lines^17^. Similarly, the present study found that MG7 induced ROS production in CRC cells in a concentration-dependent manner and NAC could prevent MG7induced cell death in HCT-116 and HT-29 cells. Moreover, pretreatment with 5 mM NAC could abolish MG7-induced apoptosis in both CRC cell lines. Taken together, these results suggest that ROS generation is involved in cytotoxic and apoptosis-inducing effects of MG7 in CRC cells. It was reported that ROS acts as an upstream signal that triggers p53 activation, resulting in the apoptosis of the CRC cells^18, 19^. In addition, ROS was found to be an important mediator for apoptosis induction in p53-independent pathway^20–22^. It was reported that doxorubicin induced apoptosis through ROS accumulation in p53-null human osteosarcoma Saos-2 cells^23^. Thus, it is likely that MG7 could induce CRC cells to undergo apoptosis via accumulation of ROS irrespective of p53 status. However, the molecular mechanism underlying these observations is still unclear.

Constitutive activation of ERK/MAPK and PI3K/AKT signaling pathways have been reported to play essential roles in CRC progression, metastasis and drug resistance^24^. By transcriptome analysis, we found that MAPK and PI3K-Akt signaling pathways mainly involved in MG7-treated CRC cells. A previous study reported that activation of MEK-ERK1/2 pathway promoted tumorigenicity and metastasis in CD133+ primary colon cancer cells and the clonogenic growth of CD133+ cells was greatly reduced by inhibiting the ERK1/2 activity^25^. Moreover, 1,4-napthoquinone and its derivatives could induce apoptosis via down-regulation of phosphorylated ERK1/2 and accumulation of ROS^22^. A recent study also demonstrated that quinalizarin exhibits apoptosis-inducing effect by suppressing ERK phosphorylation in CRC cells^26^. Remarkably, the results in this study revealed that MG7 inhibited ERK1/2 phosphorylation in HCT-116 cells, by hydrophobically binding to the ATP-binding pocket of ERK1, especially at the catalytic Lys48 residue^27^, but induced ERK1/2 phosphorylation in HT-29 cells. There is an accumulating evidence that activation of ERK1/2 generally promotes cell survival, but under certain condition, aberrant ERK1/2 activation enhances apoptosis^28, 29^. Gulati et al. revealed that TPA-modulated RAS/ERK signaling pathways work differently depending on the status of p53 in the cells^30^. Moreover, it was shown that piperlongumine induced ERK phosphorylation, leading to cell death in p53 mutant HT-29 cells^31^. Similarly, phenethyl isothiocyanate-induced apoptosis in p53-deficient PC-3 human prostate cell line was mediated via ERK activation and apoptosis-inducing effect of PEITC was abolished in the presence of an ERK1/2 inhibitor^32^. Taken together, it is likely that ERK activation may be associated with MG7-induced apoptotic cell death in p53-mutant HT-29 cells observed in this study. Notably, accumulating evidence has demonstrated that mutation of BRAF can constitutively activate MAPK/ERK signaling pathway^33–35^. Thus, it is also possible that binding of MG7 caused inhibition of ERK in BRAF wild-type HCT116 cells, but not BRAF mutant HT29 cells^36^. Further mechanistic studies need to be performed in order to gain more insight into the effects of MG7 on the ERK/MAPK signaling pathway in CRC.

Aside from ERK/MAPK signaling pathway, PI3K/AKT signaling pathway is involved in regulation of apoptosis in CRC cells, suggesting that using an agent targeting this pathway may be an effective strategy for CRC therapy. A previous study demonstrated that ramentaceone, a naphthoquinone derived from *Drosera* sp., induced apoptosis by inhibiting the PI3K/AKT pathway in breast cancer^12^. Similarly, furano-1,2-naphthoquinone isolated from Avicennia marina inhibited AKT phosphorylation, resulting in cell cycle arrest at G2/M phase and apoptosis in human oral squamous carcinoma cancer cells^37^. In addition, acetylshikonin could inhibit PI3K/AKT signaling pathway, leading to inhibition of cell proliferation and induction of cell cycle arrest at G0/G1 phase; apoptosis was observed in vitro and in vivo, the cancer cells’ growth was suppressed^38^. These findings are consistent with the results obtained in this study that MG7 effectively inhibited AKT phosphorylation in HCT-116 and HT-29 cells in a concentration dependent manner by binding to the ATP-binding pocket of AKT via hydrophobic forces and pi-sulfur interactions at Met281 residue, which is an essential interaction for kinase inhibitory activity of AKT inhibitors^39, 40^. The present study also showed that MG7 could bind to the ATPase domain of DNA gyrase, an essential bacterial enzyme involved in the DNA replication process^9^. Taken together, we propose that MG7 can be used to treat bacterial infection and cancer.

In conclusion, the present study clearly demonstrated that MG7, allyl ether of mansonone G, exerted a potent anticancer activity against p53 wild-type HCT-116 and p53 mutant HT-29 cell lines. MG7 induced ROS generation, leading to apoptosis in both HCT-116 and HT-29 cells. Western blot analysis revealed that MG7 downregulated the expression of Bcl-2 and Bcl-xL proteins in both CRC cells and upregulated the expression of BAK protein in HT-29 cells. Furthermore, MG7 downregulated AKT signaling pathway and modulated ERK1/2 signaling pathway by inhibiting ERK1/2 phosphorylation in HCT-116 cells and activating ERK1/2 phosphorylation in HT-29 cells. Molecular docking simulation predicted that MG7 could bind to the ATP-binding pocket of AKT and ERK1 via hydrophobic interactions. Taken together, the results from this study suggest that MG7 can potentially be developed as a novel anticancer agent. However, further elucidation and verification of these observations both in vitro and in vivo are warranted.

### Materials and methods

### Cell culture

Human colon carcinoma cell lines, HCT-116 and HT-29, were obtained from the American Type Culture Collection (ATCC, Manassas, VA, USA). PCS201-010 normal human dermal fibroblasts were obtained from the Faculty of Pharmacy, Rangsit University. CRL-1790 normal human colon epithelial cells were obtained from Dr. Amornpun Sereemaspun (Department of Anatomy, Faculty of Medicine, Chulalongkorn University). HCT-116 cells were maintained in RPMI-1640 (Gibco, Grand Island, NY, USA) supplemented with 10% fetal bovine serum (FBS; Gibco), 100 U/mL penicillin and 100 µg/mL streptomycin (Gibco). HT-29 cells were cultured in DMEM (Gibco) containing 10% FBS, 100 U/mL penicillin and 100 µg/mL streptomycin. PCS201-010 and CRL-1790 cells were maintained in DMEM containing 10% FBS, 100 U/mL penicillin, 100 µg/mL streptomycin and 4.5 g/L glucose. The cells were grown at 37 °C in a humidified 5% CO_2_ incubator.

### Preparation of MG and its derivatives stock solution

The heartwoods of *Mansonia gagei* was collected from Saraburi province. The identity of this plant was compared with a voucher specimen No. 43281 at the herbarium of the Royal Foresty Department of Thailand. MG was obtained by extraction of this plant heartwood by ethyl acetate. The crude extract was further purified to achieve mansonone G as described in a previous study^3^. All derivatives were semi-synthesized, purified and well-characterized by Chavasiri and colleagues^3^. All methods were performed in accordance with relevant institutional, national, and international guidelines and legislation. A 50 mM stock solutions of MG and its derivatives were prepared in dimethyl sulfoxide (DMSO; Sigma-Aldrich, St. Louis, MO, USA). In the experiments, the stock solution was diluted in culture medium to reach the appropriate final concentrations. A final concentration of 0.2% DMSO was used as a vehicle control.

### Cell viability assay

To screen for the formazan product, HCT-116 and HT-29 cells were seeded at a density of 5 × 10^4^ cells/mL in 96 well-plates and incubated overnight. The cells were treated with MG, its derivatives or oxaliplatin (positive control) at concentrations of 0.1, 1, 10 or 100 µM or 0.2% DMSO (vehicle control) in complete medium for 48 h. Subsequently, the MTT solution (0.5 mg/mL) was added and incubated for another 4 h. The medium was then removed and 150 µL of 0.2% DMSO was added to solubilize formazan crystals. Finally, the absorbance of formazan product was measured at 570 nm using a microplate reader (Thermo Fisher Scientific, Vantaa, Finland).

As for the toxicity screening, PCS201-010 cells were seeded at a density of 5 × 10^4^ cell/mL in 96-well plates overnight and then treated with MG3, MG4, MG7 or MG10 at 1, 10 and 100 µM, respectively, for 48 h. CRL-1790 cells were treated with MG7 at 1.25, 2.5, 5, 10 and 20 µM for 48 h. After incubation, MTT assays were performed as previously described^41^.

### Apoptosis assay

HCT-116 and HT-29 cells were seeded at a density of 5 × 10^4^ cells/mL in 6 well-plates and incubated overnight. HCT-116 cells were treated with MG7 at 2.5, 5 and 10 µM whereas HT-29 cells were treated with MG7 at 5, 10 and 20 µM. After 24 h incubation, the cells were collected by trypsinization and the cell pellet was incubated with 3 µL of annexin V-FITC (Invitrogen, Carlsbad, CA, USA) and 1 µL of PI (Santa Cruz Biotechnology, Dallas, TX, USA) at room temperature in the dark for 15 min. The stained cells were then analyzed using flow cytometer (BD Biosciences, Heidelberg, Germany).

### DCFH_2_-DA assay

HCT-116 and HT-29 cells were seeded at a density of 5 × 10^4^ cells/mL in 96-well plates and incubated overnight. The cells were incubated with 100 µL of 10 µM DCFH_2_-DA in Hank’s buffered salt solution (HBSS) at 37 °C in the dark for 30 min. HCT-116 cells were then treated with MG7 at 2.5, 5 and 10 µM while HT-29 cells were treated with MG7 at 5, 10 and 20 µM. After 24 h incubation, the cells were lysed with 200 µL of 1% triton-X in 0.3 NaOH. The fluorescence intensity was measured at an excitation wavelength of 485 nm and an emission wavelength of 535 nm using a fluorescence microplate reader (Thermo Fisher Scientific, Vantaa, Finland).

### RNA preparation and RNA-seq analysis

HCT-116 and HT-29 cells cancer cell lines were treated with MG7 at 5 µM and 10 µM, respectively in a humidified atmosphere at 37 °C and 5% CO_2_ for 24 h. Control cells were treated with culture medium containing solvent (0.2% DMSO). At the end of treatment cells were harvested by trypsinization. After washing in PBS, total RNA samples were isolated from the treated cells using TRIzol reagent following the recommendations of the manufacturer. The quality and quantity of RNA were analyzed using 1% agarose gel electrophoresis and NanoDrop, respectively. The extracted RNA was stored at − 80 °C until sequencing.

RNA-seq was performed using Illumina Hiseq2500 by outsource (Macrogen, Korea). The RNA-seq libraries were constructed using Illumina TruSeq RNA Library Prep Kit. One hundred fifty-bp, pair-end reads were generated in FASTQ formats of MG7 -treated HCT-116 and HT-29 raw sequence datasets. RNA-seq analysis was performed using open-source tool (Galaxy Europe). The quality of PE reads was determined using FastQC. The adapter sequences were removed and trimmed using initial Illumina Clip. The sequences were trimmed using Trimmomatic. The high-quality reads were aligned with the human genome hg38 using Hisat 2 with default parameters. Subsequently, Stringtie with Gencode V32 annotation was employed to identify and quantify the expression levels of transcripts from the preprocessed RNA-Seq alignment-assembly. The high-quality mapped transcripts were used to merge the sequencing data of control and MG7 treated groups using Stringtie Merge. Merged transcripts from control and MG7-treated groups were compared using Deseq 2 to determine the differential expression levels of given transcripts including the measure of statistical significance of the differences (the *p*-value of the test statistic and the False Discovery Rate-adjusted *p*-value of the test statistic).

### Mapping identification of DEGs and functional enrichment analysis

List of differentially expressed genes (DEG) was created only from transcripts mapped with annotated gene ID using AnnotateDEseq 2. Upregulated genes were considered those with Log2FC > 1 and p value < 0.05 and down-regulated with log2FC <  − 1 and *p* value < 0.05. Of the predicted 2486 genes and 2425 genes were differentially expressed in HCT-116 (142 up-regulated and 175 down-regulated) and in HT-29 (146 up-regulated and 129 down-regulated), respectively. To examine the biological function in which the DEGs were enriched, the DEGs between the control and MG7-treated groups were subjected to functional GO:BP (biological pathway) and KEGG pathway analyses using g: Profiler tool. Homo sapiens Orthology database was used as the reference for pathway mapping.

### Western blot

CRC cells were seeded at a density of 5 × 10^4^ cells/mL in 6 well-plates and incubated overnight. HCT-116 cells were treated with MG7 at 2.5, 5 and 10 µM while HT-29 cells were treated with MG7 at 5, 10 and 20 µM. After 24 h incubation, the treated cells were lysed with RIPA lysis buffer (Thermo Fisher Scientific, Waltham, MA, USA). The protein concentration of the cell lysate was determined using the Bio-Rad DC Protein assay reagents (Bio-Rad, Hercules, CA, USA). Twenty micrograms of protein lysate were separated on an 8% sodium dodecyl sulfate polyacrylamide gel electrophoresis (SDS-PAGE) and then transferred to a PVDF membrane. The membrane was blocked in 3% non-fat dry milk (NFDM) and incubated with antibodies against BAK, BAX, Bcl-2, Bcl-xL, cleaved caspase-3, cleaved PARP, phospho-p44/42 MAPK (ERK1/2), p44/42 MAPK (ERK1/2), phospho-Akt, Akt or GAPDH (Cell Signaling Technology, Santa Cruz, CA, USA) at 4 °C overnight. The membrane was then incubated with horseradish peroxidase (HRP)-conjugated secondary antibody (Cell signaling) at room temperature for 2 h. Protein bands were detected using chemiluminescence detection system (Thermo Fisher Scientific, Waltham, MA, USA) and analyzed using Image Studio software version 5.2 (LI-COR, Lincoln, NE, USA).

### Molecular docking

Crystal structures of human AKT (PDB ID: 4GV1)^42^ and ERK1 (PDB ID: 2ZOQ)^43^ were obtained from Protein Data Bank (PDB). SWISS-MODEL server^44^ was used to complete missing residues of the protein structures. The protonation state of all ionizable amino acids of AKT and ERK1 was characterized at pH 7.0^7^ using H++ server^45^. The structure of MG7 ligand was constructed using GaussView 5 program^46^. Molecular docking simulation of MG7 against AKT and ERK1 proteins was performed by Autodock Vina^47^ using a grid box of 15 Å × 15 Å × 15 Å with a grid spacing of 1 Å. All graphical presentations of the docked complexes were illustrated using Discovery Studio Visualizer version v19.1.0.18287 (BIOVIA, San Diego, CA, USA).

### Statistical analysis

All results were expressed as means ± standard error of mean (SEM) from at least three independent experiments performed in triplicate. Multiple comparisons were performed by a one-way analysis of variance (ANOVA) followed by LSD post hoc test. Student’s t-test was used to detect significant differences between the two groups using SPSS software. *P* value < 0.05 was considered to be statistically significant.

## Supplementary Information

Supplementary Table S1.

Supplementary Table S2.

Supplementary Table S3.

Supplementary Table S4.

Supplementary Table S5.

Supplementary Table S6.

## Data Availability

All data generated or analyzed during this study are included in this published article (and its supplementary information files).
