# Supplementary material for: Allyl ether of mansonone G as a potential anticancer agent for colorectal cancer
Source: Sci Rep. 2022 Nov 16;12:19668. doi: 10.1038/s41598-022-23997-x (PMC9668903; doi:10.1038/s41598-022-23997-x)
Supplement: Supplementary file 1 — Supplementary Table S1. [file 41598_2022_23997_MOESM1_ESM.docx]

**Table S1** Top 20 (up and down-regulated) differentially expressed genes (DEGs) between control and MG-7 treatment groups in HCT-116 cells.

| **Gene**  **name** | **GENE ID** | **Full gene name** | **Log2FC** |
| --- | --- | --- | --- |
| GNE | ENSG00000159921 | Glucosamine (UDP-N-acetyl)-2-epimerase/N-acetylmannosamine kinase | 6.212062493 |
| ANXA7 | ENSG00000138279 | Annexin A7 | 5.081586877 |
| PRSS3 | ENSG00000010438 | Serine protease 3 | 4.460606345 |
| SNRPB | ENSG00000125835 | Small nuclear ribonucleoprotein polypeptides B and B1 | 4.333069477 |
| HLA-A | ENSG00000206503 | Major histocompatibility complex, class I, A | 4.243543691 |
| TPM4 | ENSG00000167460 | Tropomyosin 4 | 4.069551518 |
| MGARP | ENSG00000137463 | Mitochondria localized glutamic acid rich protein | 4.061435134 |
| ST3GAL2 | ENSG00000157350 | ST3 beta-galactoside alpha-2,3-sialyltransferase 2 | 3.632867354 |
| ZNF619 | ENSG00000177873 | Zinc finger protein 619 | 3.553380291 |
| ZNF84 | ENSG00000198040 | Zinc finger protein 84 | 3.538777042 |
| ZNF114 | ENSG00000178150 | Zinc finger protein 114 | 3.51696013 |
| ATR | ENSG00000175054 | ATR serine/threonine kinase | 3.474303574 |
| DUSP4 | ENSG00000120875 | Dual specificity phosphatase 4 | 3.469187058 |
| PRELID3A | ENSG00000141391 | PRELI domain containing 3A | 3.468535563 |
| TSPAN1 | ENSG00000117472 | Tetraspanin 1 | 3.251628869 |
| TOMM40L | ENSG00000158882 | Translocase of outer mitochondrial membrane 40 like | 3.175397734 |
| LPIN1 | ENSG00000134324 | Lipin 1 | 2.992448294 |
| PLAT | ENSG00000104368 | Plasminogen activator, tissue type | 2.881037358 |
| ZFP90 | ENSG00000184939 | ZFP90 zinc finger protein | 2.497582603 |
| ACSS2 | ENSG00000131069 | Acyl-CoA synthetase short chain family member 2 | 2.421124415 |
| MSRB3 | ENSG00000174099 | Methionine sulfoxide reductase B3 | -1.42830137 |
| PEX10 | ENSG00000157911 | Peroxisomal biogenesis factor 10 | -1.435082232 |
| EVI5L | ENSG00000142459 | Ecotropic viral integration site 5 like | -1.586744896 |
| PLD6 | ENSG00000179598 | Phospholipase D family member 6 | -1.604228362 |
| TTC3 | ENSG00000182670 | Tetratricopeptide repeat domain 3 | -1.604837861 |
| STAT6 | ENSG00000166888 | Signal transducer and activator of transcription 6 | -1.615900161 |
| NEDD1 | ENSG00000139350 | NEDD1 gamma-tubulin ring complex targeting factor | -1.646556935 |
| SAP25 | ENSG00000205307 | Sin3A associated protein 25 | -1.888200234 |
| TMEM67 | ENSG00000164953 | Transmembrane protein 67 | -2.096336454 |
| ACBD4 | ENSG00000181513 | Acyl-CoA binding domain containing 4 | -2.138926783 |
| TMPRSS9 | ENSG00000178297 | Transmembrane serine protease 9 | -2.207130879 |
| STAG3 | ENSG00000066923 | Stromal antigen 3 | -2.357485247 |
| ZNF3 | ENSG00000166526 | Zinc finger protein 3 | -2.419874506 |
| UBD | ENSG00000213886 | Ubiquitin D | -2.661761864 |
| CRY2 | ENSG00000121671 | Cryptochrome circadian regulator 2 | -3.939214668 |
| NTN1 | ENSG00000065320 | Netrin 1 | -3.970173366 |
| RNF115 | ENSG00000265491 | Ring finger protein 115 | -4.529226701 |
| TBC1D3L | ENSG00000274512 | TBC1 domain family member 3L | -5.439916025 |
| SIRPA | ENSG00000198053 | Signal regulatory protein alpha | -5.468987595 |
| ZC3H18 | ENSG00000158545 | Zinc finger CCCH-type containing 18 | -5.567266083 |
