# Supplementary material for: Allyl ether of mansonone G as a potential anticancer agent for colorectal cancer
Source: Sci Rep. 2022 Nov 16;12:19668. doi: 10.1038/s41598-022-23997-x (PMC9668903; doi:10.1038/s41598-022-23997-x)
Supplement: Supplementary file 2 — Supplementary Table S2. [file 41598_2022_23997_MOESM2_ESM.docx]

**Table S2** Top 20 (up and down-regulated) differentially expressed genes (DEGs) between control and MG-7 treatment groups in HT-29 cells.

| **Gene name** | **GENE ID** | **Full gene name** | **Log2FC** |
| --- | --- | --- | --- |
| NMT1 | ENSG00000136448 | N-myristoyltransferase 1 | 7.799082384 |
| DENND1A | ENSG00000119522 | DENN domain containing 1A | 5.92533894 |
| E2F7 | ENSG00000165891 | E2F transcription factor 7 | 5.298820599 |
| PSD4 | ENSG00000125637 | Pleckstrin and Sec7 domain containing 4 | 5.094357516 |
| IL32 | ENSG00000008517 | Interleukin 32 | 5.08544311 |
| RPP14 | ENSG00000255154 | Ribonuclease P/MRP subunit p14 | 4.945136106 |
| XRCC4 | ENSG00000152422 | X-ray repair cross complementing 4 | 4.360057867 |
| IL36B | ENSG00000136696 | Interleukin 36 beta | 4.188974592 |
| PPP1R21 | ENSG00000162869 | Protein phosphatase 1 regulatory subunit 21 | 3.999276636 |
| GULP1 | ENSG00000144366 | GULP PTB domain containing engulfment adaptor 1 | 3.692216705 |
| ZNF710 | ENSG00000140548 | Zinc finger protein 710 | 3.688258074 |
| CHMP3 | ENSG00000115561 | Charged multivesicular body protein 3 | 3.394947079 |
| NIT1 | ENSG00000158793 | Nitrilase 1 | 3.283868069 |
| INSIG1 | ENSG00000186480 | Insulin induced gene 1 | 2.888225479 |
| ATG9B | ENSG00000181652 | Autophagy related 9B | 2.760952675 |
| NCAPD3 | ENSG00000151503 | Non-SMC condensin II complex subunit D3 | 2.667446662 |
| SDSL | ENSG00000139410 | Serine dehydratase like | 2.448896493 |
| FLOT1 | ENSG00000137312 | Flotillin 1 | 2.409207778 |
| CKAP5 | ENSG00000175216 | Cytoskeleton associated protein 5 | 2.400049694 |
| PARP10 | ENSG00000178685 | Poly(ADP-ribose) polymerase family member 10 | 2.383777134 |
| LAMA5 | ENSG00000130702 | Laminin subunit alpha 5 | -1.562285003 |
| PRDM15 | ENSG00000141956 | PR/SET domain 15 | -1.613710096 |
| MAST1 | ENSG00000105613 | Microtubule associated serine/threonine kinase 1 | -1.652341402 |
| CASKIN2 | ENSG00000177303 | CASK interacting protein 2 | -1.724696578 |
| PADI4 | ENSG00000159339 | Peptidyl arginine deiminase 4 | -1.788910545 |
| ZNF544 | ENSG00000198131 | Zinc finger protein 544 | -1.793883969 |
| KLRK1 | ENSG00000213809 | Killer cell lectin like receptor K1 | -1.886690631 |
| ARTN | ENSG00000117407 | Artemin | -1.97863782 |
| KCNIP3 | ENSG00000115041 | Potassium voltage-gated channel interacting protein 3 | -2.399155547 |
| PLXNA3 | ENSG00000130827 | Plexin A3 | -2.399575638 |
| EXOC3 | ENSG00000180104 | Exocyst complex component 3 | -2.454326145 |
| INTS1 | ENSG00000164880 | Integrator complex subunit 1 | -2.501096649 |
| OSBPL7 | ENSG00000006025 | Oxysterol binding protein like 7 | -3.07988772 |
| HEXIM1 | ENSG00000186834 | HEXIM P-TEFb complex subunit 1 | -3.131014777 |
| ZNF169 | ENSG00000175787 | Zinc finger protein 169 | -3.643912359 |
| ZNF10 | ENSG00000256223 | Zinc finger protein 10 | -4.091162039 |
| GRSF1 | ENSG00000132463 | G-rich RNA sequence binding factor 1 | -4.253071929 |
| NUDT4 | ENSG00000173598 | Nudix hydrolase 4 | -4.28467823 |
| COL4A6 | ENSG00000197565 | Collagen type IV alpha 6 chain | -4.534257645 |
| MAML1 | ENSG00000161021 | Mastermind like transcriptional coactivator 1 | -7.216395149 |
