# Supplementary material for: Allyl ether of mansonone G as a potential anticancer agent for colorectal cancer
Source: Sci Rep. 2022 Nov 16;12:19668. doi: 10.1038/s41598-022-23997-x (PMC9668903; doi:10.1038/s41598-022-23997-x)
Supplement: Supplementary file 3 — Supplementary Table S3. [file 41598_2022_23997_MOESM3_ESM.docx]

**Table S3** Functional enrichment analysis of DEGs between control and MG 7 treatment in CRC cells by GO: B.P (biological process) in HCT-116 cells

| **Biological function** | **GO:BP ID** | **Intersections** | **Focus genes** |
| --- | --- | --- | --- |
| ***Up-regulated genes*** | | | |
| Apoptotic process | GO:0006915 | 9 | APC,CYLD,HNRNPK,MELK,NEURL1,PDPK1,PTPRH,SLC39A10,ZNF385B |
| Cell cycle | GO:0007049 | 16 | APC,ATR,BOD1L1,CDK7,CDK8,CYLD,JUND,KIF13A,LPIN1,MELK,NDEL1,SEPTIN7,SPTBN1,SUN1,TP53I13,UHRF2 |
| ***Down-regulated genes*** | | | |
| Apoptotic process | GO:0006915 | 12 | GSK3A,HELLS,HTT,IKBKG,LMBR1L,NDUFA13,NOL3,NTN1,PAX8,THOC1,TXNDC5,UBD |
| Cell cycle | GO:0007049 | 20 | BTRC,CCNT2,DSN1,HELLS,HTT,LIG1,MIS18BP1,MYO19,NEDD1,PKD2,PLD6,RMDN1,RTEL1,STAG3,SUN2,THOC1,TMEM67,TTC19,TUBG2,UBD |
