# Supplementary material for: Allyl ether of mansonone G as a potential anticancer agent for colorectal cancer
Source: Sci Rep. 2022 Nov 16;12:19668. doi: 10.1038/s41598-022-23997-x (PMC9668903; doi:10.1038/s41598-022-23997-x)
Supplement: Supplementary file 4 — Supplementary Table S4. [file 41598_2022_23997_MOESM4_ESM.docx]

**Table S4** Functional enrichment analysis of DEGs between control and MG7 treatment in CRC cells by KEGG in HCT-116 cells

| **Molecular pathway** | **KEGG ID** | **Intersections** | **Focus genes** |
| --- | --- | --- | --- |
| ***Up-regulated genes*** | | | |
| MAPK signaling pathway | KEGG:04010 | 4 | DUSP4,JUND,MAP4K2,NFKB2 |
| Cell cycle | KEGG:04110 | 2 | ATR,CDK7 |
| mTOR signaling pathway | KEGG:04150 | 2 | LPIN1,PDPK1 |
| Necroptosis | KEGG:04217 | 2 | CYLD,HIST1H2AC |
| NF-kappa B signaling pathway | KEGG:04064 | 2 | CYLD,NFKB2 |
| PPAR signaling pathway | KEGG:03320 | 2 | ACSL1,PDPK1 |
| Ras signaling pathway | KEGG:04014 | 2 | ABL2,RALGAPA2 |
| Glutathione metabolism | KEGG:00480 | 1 | GGT7 |
| p53 signaling pathway | KEGG:04115 | 1 | ATR |
| Apoptosis | KEGG:04210 | 1 | PDPK1 |
| PI3K-Akt signaling pathway | KEGG:04151 | 1 | PDPK1 |
| Phosphatidylinositol signaling system | KEGG:04070 | 1 | MTMR14 |
| TGF-beta signaling pathway | KEGG:04350 | 1 | THSD4 |
| ***Down-regulated genes*** | | | |
| PI3K-Akt signaling pathway | KEGG:04151 | 2 | EPOR,IKBKG |
| MAPK signaling pathway | KEGG:04010 | 2 | IKBKG,PRKACB |
| p53 signaling pathway | KEGG:04115 | 1 | ADGRB1 |
| Apoptosis | KEGG:04210 | 1 | IKBKG |
| Phosphatidylinositol signaling system | KEGG:04070 | 1 | DGKH |
| NF-kappa B signaling pathway | KEGG:04064 | 1 | IKBKG |
