# Supplementary material for: Allyl ether of mansonone G as a potential anticancer agent for colorectal cancer
Source: Sci Rep. 2022 Nov 16;12:19668. doi: 10.1038/s41598-022-23997-x (PMC9668903; doi:10.1038/s41598-022-23997-x)
Supplement: Supplementary file 5 — Supplementary Table S5. [file 41598_2022_23997_MOESM5_ESM.docx]

**Table S5** Functional enrichment analysis of DEGs between control and MG 7 treatment in CRC cells by GO: B.P (biological process) in HT-29 cells

| **Biological function** | **GO:BP ID** | **Intersections** | **Focus genes** |
| --- | --- | --- | --- |
| ***Up-regulated genes*** | | | |
| Apoptotic process | GO:0006915 | 7 | CHMP3,GULP1,ITPR1,MAPK8,NMT1,PAEP,PIK3CD |
| Cell cycle | GO:0007049 | 14 | ACTR5,ARL3,CEP152,CHMP3,CKAP5,DBF4,E2F7,EPB41,NCAPD3,PLD6,TACC1,TDRKH,TEX15,TPRA1 |
| ***Down-regulated genes*** | | | |
| Apoptotic process | GO:0006915 | 13 | ARAF,DMPK,FRS2,GABARAP,IL7R,IRF7,KCNIP3,MAZ,PTPN1,SGMS1,SMAD6,TMBIM1,ZMYND11 |
| Cell cycle | GO:0007049 | 7 | DSN1,EML1,HEXIM1,KIF25,OBSL1,SPECC1L,ZMYND11 |
