# Supplementary material for: Allyl ether of mansonone G as a potential anticancer agent for colorectal cancer
Source: Sci Rep. 2022 Nov 16;12:19668. doi: 10.1038/s41598-022-23997-x (PMC9668903; doi:10.1038/s41598-022-23997-x)
Supplement: Supplementary file 6 — Supplementary Table S6. [file 41598_2022_23997_MOESM6_ESM.docx]

**Table S6** Functional enrichment analysis of DEGs between control and MG7 treatment in CRC cells by KEGG in HT-29 cells

| **Molecular pathway** | **KEGG ID** | **Intersections** | **Focus genes** |
| --- | --- | --- | --- |
| ***Up-regulated genes*** | | | |
| Apoptosis | KEGG:04210 | 3 | ITPR1,MAPK8,PIK3CD |
| Ras signaling pathway | KEGG:04014 | 2 | MAPK8,PIK3CD |
| Necroptosis | KEGG:04217 | 2 | CHMP3,MAPK8 |
| Phosphatidylinositol signaling system | KEGG:04070 | 2 | ITPR1,PIK3CD |
| mTOR signaling pathway | KEGG:04150 | 1 | PIK3CD |
| MAPK signaling pathway | KEGG:04010 | 1 | MAPK8 |
| Cell cycle | KEGG:04110 | 1 | DBF4 |
| PI3K-Akt signaling pathway | KEGG:04151 | 1 | PIK3CD |
| ***Down-regulated genes*** | | | |
| PI3K-Akt signaling pathway | KEGG:04151 | 6 | COL4A6,G6PC3,IL4R,IL7R,LAMA5,TNC |
| MAPK signaling pathway | KEGG:04010 | 2 | ARAF,NFKB2 |
| JAK-STAT signaling pathway | KEGG:04630 | 2 | IL4R,IL7R |
| Apoptosis | KEGG:04210 | 1 | SPTAN1 |
| NF-kappa B signaling pathway | KEGG:04064 | 1 | NFKB2 |
| mTOR signaling pathway | KEGG:04150 | 1 | WDR59 |
| ErbB signaling pathway | KEGG:04012 | 1 | ARAF |
